# Oral Administration of Recombinant *Lactococcus lactis* Expressing HSP65 and Tandemly Repeated P277 Reduces the Incidence of Type I Diabetes in Non-Obese Diabetic Mice

**DOI:** 10.1371/journal.pone.0105701

**Published:** 2014-08-26

**Authors:** Yanjun Ma, Jingjing Liu, Jing Hou, Yuankai Dong, Yong Lu, Liang Jin, Rongyue Cao, Taiming Li, Jie Wu

**Affiliations:** 1 Forensic Center, Nanjing Forest Police College, Nanjing, People's Republic of China; 2 Minigene Pharmacy Laboratory, School of life Science and Technology, China Pharmaceutical University, Nanjing, People's Republic of China; La Jolla Institute for Allergy and Immunology, United States of America

## Abstract

Diabetes mellitus type 1 (DM1) is an autoimmune disease that gradually destroys insulin-producing beta-cells. We have previously reported that mucosal administration of fusion protein of HSP65 with tandem repeats of P277 (HSP65-6P277) can reduce the onset of DM1 in non-obese diabetic (NOD) mice. To deliver large amounts of the fusion protein and to enhance long-term immune tolerance effects, in the present study, we investigated the efficacy of using orally administrated *L. lactis* expressing HSP65-6P277 to reduce the incidence of DM1 in NOD mice. *L. lactis* strain NZ9000 was engineered to express HSP65-6P277 either constitutively or by nisin induction. After immunization via gavage with the recombinant *L. lactis* strains to groups of 4-week old female NOD mice for 36 weeks, we observed that oral administration of recombinant *L. Lactis* resulted in the prevention of hyperglycemia, improved glucose tolerance and reduced insulitis. Immunologic analysis showed that treatment with recombinant *L. lactis* induced HSP65- and P277- specific T cell immuno-tolerance, as well as antigen-specific proliferation of splenocytes. The results revealed that the DM1-preventing function was in part caused by a reduction in the pro-inflammatory cytokine IFN-γ and an increase in the anti-inflammatory cytokine IL-10. Orally administered recombinant *L. lactis* delivering HSP65-6P277 may be an effective therapeutic approach in preventing DM1.

## Introduction

Diabetes mellitus type I (DM1), also known as insulin-dependent diabetes mellitus (IDDM), is a chronic autoimmune disease clinically characterized by hyperglycemia, resulting from the destruction of insulin-producing pancreatic β cells due to autoreactive T cells. Different therapeutic approaches have been exploited aimed at regulating this autoimmune response, including broad immunosuppressive drugs, antibody-based immunotherapies and antigen-based immunotherapy [1], [2]. However, the substantial short- and long-term toxic effects of immunosuppressive agents have blocked the adoption of nonspecific immunosuppression into clinical practice [3]. Antibody-based immunotherapies failed to discriminate between autoreactive versus non-autoimmune effectors. Currently, more attention has been given to antigen-based immunotherapies that allow the selective targeting of disease-relevant T cells, while leaving the remainder of the immune system intact. Heat-shock protein (HSP) is an important self-antigen in the pathogenesis of diabetes. DiaPep277 is a 24 amino-acid peptide derived from human HSP60 that has been demonstrated to modulate immunological attack on β cells in NOD mouse model of DM1 [4]. Results from a double-blind phase 3 clinical trial also showed that DiaPep277 safely contributes to preserve of β cell function and improve glycemic control in DM1 patients [5]. In our previous study, we have reported that nasal administration of P277 repeat sequences using HSP65 as an immunogenic carrier (HSP65-6P277) significantly decreased the incidence of diabetes and inhibited insulitis in NOD mice [6]. However, due to the proteolytic self-degradation nature of HSP65 [7], HSP65-6P277 is not stable through the process of isolation, purification and storage (data unpublished). In addition, conventional immunization methods, such as intramuscular injection, subcutaneous injection and intranasal administration, are not sufficient or suitable to deliver large quantities of peptides or long-term treatments, especially for children and elderly people. New vaccination methods are needed to meet these clinical requirements.

The use of microbial-based vaccines is a novel area of research that holds great promise. Different living lactic acid bacteria strains, including *Lactococcus lactis* (*L. lactis*) [8], [9], were shown to stimulate non-specific immune responses from human peripheral blood mononuclear cells [10]. Genetically modified *L. lactis* has been used for intestinal delivery of heterologous antigens [11], human anti-inflammative cytokine [12], [13], and DM1 related protein such as glutamic acid decarboxylase (GAD)-65 for antigen-specific tolerance induction [14], [15], thus providing a novel strategy for the treatment of autoimmune, inflammatory, and allergic gastrointestinal diseases.

In the present study, we report that oral administration of recombinant *L. lactis* strains that express HSP65-6P277 can successfully reduce the onset of DM1 in NOD mice. The results further illustrate the potential utility of genetically modified *L. lactis* as a safe and effective vaccine delivery vector to elicit antigen-specific tolerance for the treatment of autoimmune diseases such as DM1.

## Methods

### Bacterial strains

*L. lactis* NZ9000 and all recombinant *L. lactis* strains were grown at 30°C in M17 medium containing 0.5% glucose (GM17) without shaking. *E. coli* DH5α was grown in aerated Luria-Bertani medium at 37°C. *L. lactis* NZ9000 and pCYT: Nuc (staphylococcal nuclease protein, Nuc) plasmid were kindly provided by Dr. Shuhua Tan (School of Life Science and Technology, China Pharmaceutical University). pET28a: HSP65-6P277 plasmid was constructed previously in our laboratory [16]. When required, kanamycin (Ka^R^) was used at a concentration of 50 µg/ml for *E. coli* while chloramphenicol (Cm^R^) was used at 10 µg/ml for *E. coli*, and at 7.5 µg/ml for *L. lactis*.

### Construction of *L. lactis* expressing HSP65-6P277

Unless otherwise indicated, all plasmid constructions were performed in *Escherichia coli* (*E. coli*) DH5α and then transferred into *L. lactis* NZ9000 by electroporation [17]. Two plasmids were constructed for the expression of HSP65-6P277. Plasmid pCYT: HSP65-6P277 expressed intracellular HSP65-6P277 inducible by nisin and plasmid pHJ: HSP65-6P277 expressed extracellular form HSP65-6P277 constitutively. The cDNA sequence of HSP65-6P277 was amplified from pET28a: HSP65-6P277 using a sense primer (5′-CCA ATG CAT CAG CCA AGA CAA TTG CGT ACG AC-3′) and an antisense primer (5′- TCG ATA TCT CCG GAT ATA GTT CCT CCT TTC AG-3′). A NisI site at the N-terminus of HSP65-6P277 and an EcoRV site at the C-terminus of HSP65-6P277 were added. The PCR-amplified product of HSP65-6P277 was purified and then digested with NisI and EcoRV.

**Figure 1:**
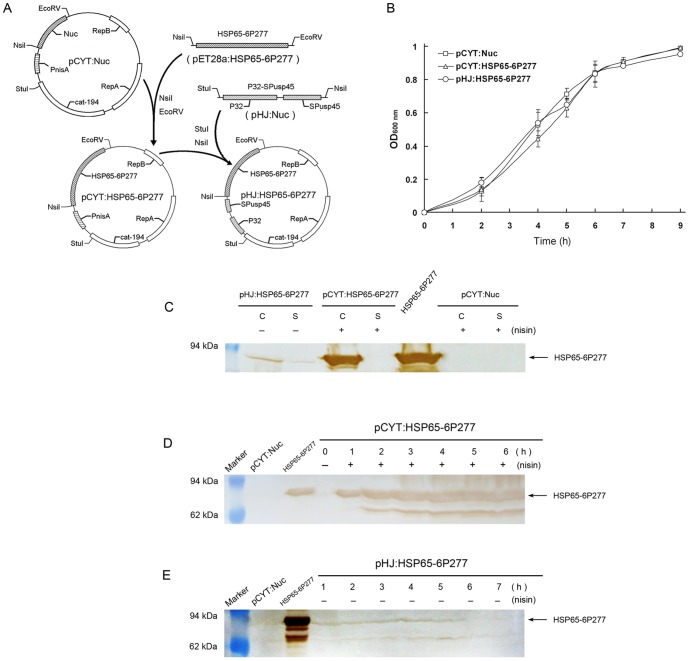
Production of HSP65-6P277 in *L. lactis*. (A) Construction of plasmids pCYT: HSP65-6P277 and pHJ: HSP65-6P277. (B) The growth curve of recombinant *L. lactis* pCYT: Nuc, *L. lactis* pCYT: HSP65-6P277, and *L. lactis* pHJ: HSP65-6P277. (C) HSP65-6P277 production in cell and supernatant fractions of recombinant *L. lactis* strains. Protein extracts were prepared and were analyzed by Western blotting. (D, E) Expression of protein HSP65-6P277 at different growth periods of recombinant *L. lactis* pCYT: HSP65-6P277 (D) and *L. lactis* pHJ: HSP65-6P277 (E), respectively. Protein extracts were prepared from cell lysates and were analyzed by Western blotting. Purified HSP65-6P277 protein served as a positive control. Triplicate experiments were performed in a parallel manner and representative immunoblots are shown. Abbreviations: C, cell lysates; S, supernatant fraction; +, induced with nisin; –, without induced.

To generate pCYT: HSP65-6P277, the vector pCYT: Nuc was also digested with NisI and EcoRV and then ligated with the double-digested PCR product of HSP65-6P277 (Fig. 1A). To construct pHJ: HSP65-6P277, we created plasmid pHJ by replacing the nisin-inducible promoter PnisA with a constitutive promoter P32 [18] (GenBank accession number M24764.1) in pCYT plasmid, and inserted a DNA fragment encoding ribosome binding site (RBSusp45) and signal peptide of Usp45 protein [19] from *L. lactis* subsp Cremoris (SPusp45, GenBank accession number M60178.1) (Fig 1A). The synthetic gene was digested with StuI and NsiI restriction endonucleases, and then ligated into StuI-NsiI-digested pCYT: Nuc. Synthesis of DNA fragments and construction of the pHJ vector was performed by Genscript Biotech, Nanjing, China [20]. All plasmids constructed were verified by DNA sequencing.

Plasmids pCYT: HSP65-6P277 and pHJ: HSP65-6P277 were further introduced into *L. lactis* NZ9000 by electrotransformation as described previously [17]. The recombinant *L. lactis* strains were named LL-pCYT: HSP65-6P277 and LL-pHJ: HSP65-6P277 respectively.

### Expression of HSP65-6P277 in *L. lactis*

Recombinant *L. lactis* strains were cultured overnight at 30°C in GM17 medium followed by inoculation at 1∶50 into fresh GM17 medium for a few hours. To measure the production of HSP65-6P277, when the optical density at 600 nm (OD_600_) was 0.4, strain LL-pCYT: HSP65-6P277 was induced by 50 ng/ml nisin (Silver-Elephant Bioengineering Co., Ltd, Zhejiang, China) when itsthe optical density at 600 nm (OD_600_) was 0.4, cultured for another 1∼8 hours and then harvested. Strain LL-pHJ: HSP65-6P277 was harvested directly without inducing.

### Western blot analysis

Western blot was performed to detect the expression of HSP65-6P277 in the recombinant *L. lactis* strains [21]. Briefly, harvested bacteria were lysed and proteins were separated by sodium dodecyl sulfate-polyacrylamide gel electrophoresis (SDS-PAGE) (10% polyacrylamide) and transferred onto nitrocellulose membranes (Millipore, USA). The membranes were blocked, washed, and probed with 1∶100 diluted mice polyclonal antibody against HSP65 for 2 h. Blots were then washed and incubated with HRP-conjugated goat anti-mouse IgG (Sigma, USA). The reaction was developed by using 0.05% (w/v) 3,3′-diaminobenzidine tetrahydrochloride and 0.012% (v/v) H_2_O_2_ for 15 min at 37°C. Purified fusion protein HSP65-6P277 served as the positive control.

### Animals

Four-week-old female NOD/Lt mice with an average weight of 20 g were purchased from Shanghai Slaccas Experiment Animal Limited Company (Shanghai China) and maintained under pathogen-free conditions during these studies. All animal protocols were reviewed and performed in compliance with the National Institutes of Health Guide for the Care and Use of Laboratory Animals and approved by the Ethics Committee on Animal Experiments of China Pharmaceutical University.

### Vaccination

NOD mice were randomly divided into four groups (n = 12 per group). The vehicle control group received phosphate-buffered saline (PBS) 200 µl via gavage and the negative control group (group pCYT: Nuc) was treated with control strain *L. lactis* NZ9000 that contains empty vector and irrelevant antigen (pCYT: Nuc). Group pCYT: HSP65-6P277 and group pHJ: HSP65-6P277 were fed with recombinant *L. lactis* strain LL-pCYT: HSP65-6P277 and LL-pHJ: HSP65-6P277, respectively.

When the OD_600_ reached 0.5, LL-pHJ: HSP65-6P277 were harvested and used for inoculation. *L. lactis* strains bearing pCYT vectors were induced by nisin for 3 h before collection and administration. Treatment group mice were orally administrated with 2×10^9^ CFU of the corresponding recombinant *L. lactis* strain in 200 µl PBS medium. All groups were gavaged once daily for the first 7 days and once every week for the following 36 weeks.

### Assessment of Diabetes

Mice were monitored monthly for the development of hyperglycemia with a Hitachi automatic analyzer (Yicheng, Beijing, China). Diabetes was characterized by weight loss and persistent hyperglycemia. Blood glucose was measured in retinal vein plexus blood and animals were considered to be diabetic when two consecutive blood glucose measurements exceeded 11 mmol/L.

### Intraperitoneal glucose tolerance test

The intraperitoneal glucose tolerance test was performed at 40 weeks of age. Glucose (1.5 g/kg) was administered intraperitoneally to conscious animals after an overnight fast. Blood samples were collected from the retinal vein plexus and the blood glucose level was measured with a glucometer (Yicheng, Beijing, China).

### Pancreas histopathology

At the end of the experimental period (41 weeks), insulitis in all groups was evaluated by histology as described previously [22]. Briefly, cervical vertebra dislocation was performed to make the mice sudden death to minimize suffering of the animals. Then, pancreata were removed and fixed with 10% formalin solution, embedded in paraffin, sections and stained with H&E (Sangon). The degree of insulitis was scored by a pathologist in a blinded fashion. Insulitis grade was determined as follows: 0 =  intact islet; 1 =  peri-insulitis; 2 =  moderate insulitis (<50% of the islet infiltrated); 3 =  severe insulitis (≥50% of the islet infiltrated) and 4 =  destructive insulitis [23], [24]. A minimum of 20 islets was scored for each pancreas.

### Splenocytes proliferation assay

The proliferation of splenocytes were determined by 3-(4,5-dimethylthiazol-2-yl)-2,5-diphenyl tetrazolium bromide (MTT) (Sigma, USA) assay. Cervical vertebra dislocation was performed. to the NOD mice, Ssplenocytes from control and treated NOD mice were isolated, diluted and re-suspended as 8×10^5^ cells per well with 0.2 ml of RPMI-1640 culture medium (Gibco, Paisley, UK) in 96-well plates. Performed in quadruplicate, splenocytes were treated with various antigens at 10 µg/ml: BSA, Con A (Sigma, USA), HSP65 and P277, respectively, and incubated in a 37°C, 5% CO_2_ incubator for 48 h. At the end of testing, 20 µl MTT solution (5 mg/ml in PBS) was added to each well and incubated for an additional 4 h at 37°C [25]. The supernatant was discarded and 100 µl DMSO (Sigma, USA) was added to dissolve the formazan crystal. The absorbance was measured at 570 nm with a 630 nm reference wave in a Universal Microplate Reader (EL800, BIO-TEK, Inc., Winooski, USA). The magnitude of the proliferative response was expressed as a stimulation index (SI) defined as the ratio of the mean absorption of cells cultured with antigen to that cultured with medium alone. Each measurement was carried out in triplicate.

### Cytokine assay

Splenocytes were isolated and cultured in RPMI 1640 medium (4×10^6^/ml), and then stimulated with 10 µg/ml of P277. Cytokine levels in culture supernatants were evaluated after 72 h. Concentrations of IL-10, IFN-γ were determined by ELISA according to the manufacturer instructions using a BioLegend ELISA system (BioLegend, San Diego, USA). Cytokine levels were calculated based on standard curves. Each sample was measured in triplicate. The minimum detectable concentrations were 31.3 pg/ml for IL-10 and 15.6 pg/ml for IFN-γ.

### Statistical analysis

The results here are presented as mean ± the standard error of the mean (SEM). Statistical comparisons of means were performed using one-way analysis of variance (ANOVA) followed by the Tukey post test. Differences of survival rates were assessed by χ^2^ analysis. Values of *P*<0.05 were considered significant.

## Results

### Production of HSP65-6P277 in *L. lactis*

Growth curves of recombinant *L. lactis* strains LL-pCYT: HSP65-6P277 and LL-pHJ: HSP65-6P277 showed that these bacterium achieved logarithmic growth at about 3∼5 h after inoculation (Fig 1B). Samples from exponential cultures of *L. lactis* were analyzed by Western blot using anti-HSP65-6P277 antibodies (Fig 1C). Both cell lysates (C) samples and culture supernatant (S) fractions from the culture medium were examined. The results showed that the vehicle control, *L. lactis* pCYT: Nuc, did not express any HSP65-6P277 neither in the cell lysate nor supernatant. LL-pCYT: HSP65-6P277 expressed the HSP65-6P277 protein only in the cell lysate but not in supernatant after nisin induction. LL-pHJ: HSP65-6P277 expressed the HSP65-6P277 protein in both the cell lysate and the culture supernatant. The peptide production by LL-pHJ: HSP65-6P277 was not as high as that from LL-pCYT: HSP65-6P277. These results indicated that antigen proteins were correctly expressed and located.

To further determine the peak expression time of HSP65-6P277 in *L. lactis* strains, cellular fractions from different periods of growth were collected and analyzed by Western blot (Fig 1D and Fig 1E). It was found that abundant cytoplasmic protein HSP65-6P277 was expressed in LL-pCYT: HSP65-6P277 right after induction with nisin and maintained with high expression level. No HSP65-6P277 expression was detected without nisin (Fig 1D). As shown in Fig. 1E, the expression level of protein HSP65-6P277 in LL-pHJ: HSP65-6P277 increased with time and reached a peak at about 4 hours (OD_600 nm_ 0.5), before gradually decreasing at the end of the logarithmic growth phase. Therefore, LL-pCYT: HSP65-6P277 was incubated with nisin for 3 h before being harvested and used for vaccination. Also, LL-pHJ: HSP65-6P277 was harvested in logarithmic growth phase for intragastric administration.

### Prevention of DM1 in NOD Mice by oral administration of recombinant *L. lactis* expressing HSP65-6P277

**Figure 2:**
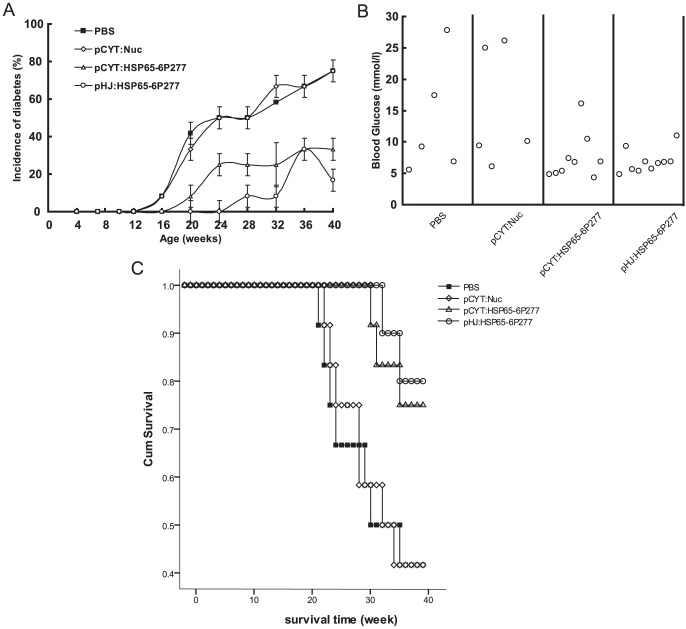
Effect of oral administration of recombinant *L. lactis* expressing HSP65-6P277 on IDDM in NOD mice. NOD mice were orally administered 2×10^9^ CFU of the corresponding recombinant *L. lactis* strain in 200 µl PBS medium, or were given 200 µl PBS medium alone, once daily in the first week and once every week in the following weeks lasting for 40 weeks. The development of diabetes was monitored until 40 weeks of age by observing the onset of hyperglycemia. (n = 12 per group) (A) The cumulative incidence of diabetes. (B) Concentration of blood glucose of NOD mice alive at the age of 41 w. (C) Survival curve of NOD mice.

The progression of diabetes in NOD mice was determined by the development of hyperglycemia and recorded in real time (Fig 2). It can be seen that, at the beginning of treatment, all animals were healthy and had normal blood glucose levels. The PBS and pCYT: Nuc vehicle control groups gradually developed diabetes over time. In both groups, 75% of the mice developed hyperglycemia within 20∼41 weeks and nearly 60% died up to 41 weeks of age (Fig 2A). In contrast, merely 25% mice in the LL-pCYT: HSP65-6P277 group developed hyperglycemia, a 67% reduction compared to the control. The survival rate was 75% in this treatment group. In the LL-pHJ: HSP65-6P277 group, the incidence of diabetes was 16.7% and the survival rate was 83.3% (Fig 2A and Fig 2C).

Among surviving NOD mice, 40% in the PBS group and the pCYT: Nuc group developed hyperglycemia (the average blood glucose level reached 22.6 mmol/l and 25.5 mmol/l, respectively). In contrast, only 11.1% in the LL-pCYT: HSP65-6P277 group developed hyperglycemia (16.1 mmol/l) and no mice in the group LL-pHJ: HSP65-6P277 were diagnosed with disease (data not shown). There was no statistical significance between the PBS group and the pCYT: Nuc group based on the incidence of diabetes and survival rate. These data demonstrate that recombinant *L. lactis* expressing HSP65-6P277 is effective at preventing the onset of DM1.

### Improved glucose tolerance in mice administered recombinant *L. lactis* expressing HSP65-6P277

**Figure 3:**
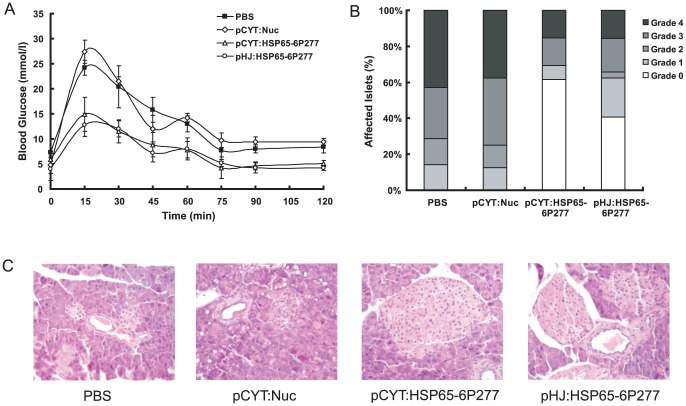
Effect of treatment with recombinant *L. lactis* expressing HSP65-6P277 on insulitis in NOD mice. (A) Improvement in glucose tolerance in NOD mice treated with recombinant *L. lactis* expressing HSP65-6P277. The intraperitoneal glucose tolerance test was performed after treatment at 40 weeks of age. All the alive mice were tested. (n = 5 in group PBS, n = 5 in group pCYT: Nuc, n = 9 in group pCYT: HSP65-6P277, and n = 10 in group pHJ: HSP65-6P277) (B) Insulitis scoring of pancreata from the different groups of treated NOD mice (n = 12 per group). At the end of the observation period, pancreata were obtained for histological examination from the different groups of treated mice. Insulitis grade was determined as follows: intact islets or prior to detectable leukocyte infiltration (grade 0); minimal peripheral infiltration or moderate peri-insular infiltration of the islets (grade 1); intraislet infiltration <50% of the islets (grade 2); extensive infiltration >50% of the islets (grade 3); much necrosis and marked atrophy of pancreas islets (grade 4). (C) Photomicrographs of representative islets from pancreas tissue of different group (original magnification, ×200) (n = 12 per group).

When comparing recombinant *L. lactis*-treated mice with control mice, weekly gavage was tolerated well in all mice and there was no difference in food intake or weight. To further detect the protective effect of oral administration of recombinant *L. lactis* expressing HSP65-6P277, we also compared the glucose tolerance. The results showed that at the end of the observation period of 41 weeks, mice treated with recombinant *L. lactis* expressing either form of HSP65-6P277 (both LL-pCYT: HSP65-6P277 and LL-pHJ: HSP65-6P277) did not exhibit a diabetic response to glucose challenge, and the glucose tolerance effect was not impaired. On the other hand, both control groups exhibited a diabetic profile as expected: the blood glucose regulation ability was weaker and the blood glucose fluctuation was comparatively larger (Fig 3A).

### Reduction of insulitis by recombinant *L. lactis* expressing HSP65-6P277

In order to understand the mechanism underlying the prevention of DM1 by recombinant *L. lactis*, we examined the progression of insulitis in NOD mice. In Fig 3B, our results showed a significantly higher percentage of intact islets (grade 0 and grade 1) in LL-pCYT: HSP65-6P277- and LL-pHJ: HSP65-6P277-treated mice compared with PBS- and vehicle-treated control mice. The parallel staining showed a greater number of lymphocytes in the intraislet infiltration around the islets and more remarkable atrophy of the pancreas islets in both control groups. In contrast, only slight peripheral infiltration and a few lymphocytes within the peri-insular infiltration of pancreas were observed in the LL-pCYT: HSP65-6P277 and LL-pHJ: HSP65-6P277 treated mice (Fig 3C). These results suggested that oral administration of recombinant *L. lactis* expressing HSP65-6P277 preserved islet integrity in NOD mice.

### Inhibition of T cell proliferation by recombinant *L. lactis* expressing HSP65-6P277

**Figure 4:**
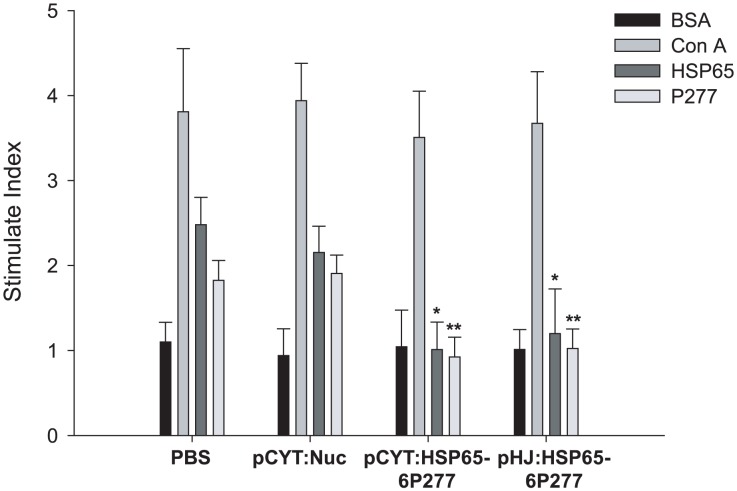
Effect of treatment with recombinant *L. lactis* expressing HSP65-6P277 on T cell proliferation. At the end of the observation period, spleens were removed and T cell proliferation was assayed in vitro using a 72 h stimulation with 10 µg/ml BSA, HSP65, P277 or the T cell mitogen Con A. Data were expressed as mean stimulation index (SI) of triplicate samples ±SEM. *p<0.05; **, *p*<0.01 compared with the PBS-treated group. SI was defined as the ratio of the mean absorption of cells cultured with antigen to the mean absorption of cells cultured with medium alone. All the alive mice were tested (n = 5 in group PBS, n = 5 in group pCYT: Nuc, n = 9 in group pCYT: HSP65-6P277, and n = 10 in group pHJ: HSP65-6P277).

To investigate whether diabetic antigen-specific tolerance was induced by recombinant *L. Lactis*, splenocyte proliferation assay was performed. As shown in Fig 4, splenocytes from all groups showed similar strong positive reactivations to the positive control ConA but not to the irrelevant protein BSA, a negative control. However, splenocytes from PBS- and vector control groups manifested spontaneous reactivity to HSP65 and P277 peptides. In contrast, splenocytes from the LL-pCYT: HSP65-6P277 and LL-pHJ: HSP65-6P277 treatment groups had significantly reduced proliferation of bulk splenocytes in response to HSP65 and P277. The stimulation index (SI) of HSP65 were 0.923±0.234 in the LL-pCYT: HSP65-6P277 group (*P* = 0.0169 compared with the PBS-treated group) and 1.199±0.524 in the LL-pHJ: HSP65-6P277group (*P* = 0.0235 compared with the PBS-treated group). No significant statistical difference was observed between the PBS-treated group and the pCYT: Nuc group (*P* = 0.379). The stimulation index (SI) of P277 was 1.012±0.322 in the LL-pCYT: HSP65-6P277 group (*P* = 0.0066 compared with the PBS-treated group) and 1.024±0.229 in the LL-pHJ: HSP65-6P277 group (*P* = 0.0023 compared with the PBS-treated group). No significant statistical difference was observed between the PBS-treated group and the pCYT: Nuc group (*P* = 0.792). These results indicate that oral administration of recombinant *L. lactis* enhanced antigen-specific immune tolerance to HSP65 and P277 peptide fragments, consequently preventing the development of diabetes.

### Effect of oral administration of *L. lactis* expressing HSP65-6P277 on cytokines

**Figure 5:**
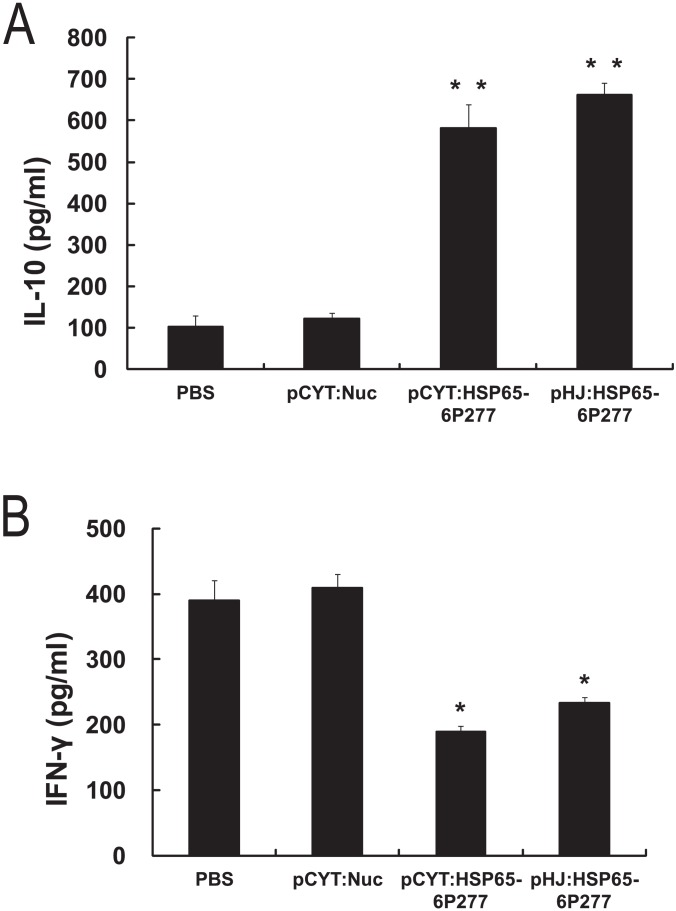
Effect of treatment with recombinant *L. lactis* expressing HSP65-6P277 on cytokine production. Splenocytes were isolated from the mice treated with PBS or recombinant *L. lactis* strains respectively containing different plasmids (pCYT: Nuc, pCYT: HSP65-6P277, or pHJ: HSP65-6P277). The splenocytes were then stimulated with 10 µg/ml of P277. Supernatants were collected after 72 h of stimulation. IL-10 and IFN-γ were quantitated in culture supernatants using an ELISA kit according to the manufacturer's instructions. Data are shown as mean ±SEM. of triplicates. * *P*<0.05; **, *P*<0.01 compared with the PBS-treated group. All the alive mice were tested (n = 5 in group PBS, n = 5 in group pCYT: Nuc, n = 9 in group pCYT: HSP65-6P277, and n = 10 in group pHJ: HSP65-6P277).

Enhanced pro-inflammatory Th1-like immune response and decreased anti-inflammatory Th2-like one have been identified in DM1 disease animal models and patients. To detect whether oral administration of recombinant *L. lactis* expressing HSP65-6P277 can enhance the Th2-like immune response, production of the cytokines IL-10 and IFN-γ was measured 72 h after stimulation with the peptide P277 in splenocytes isolated from control and treatment groups. As shown in Fig 5A, IL-10 production was significantly higher in both the LL-pCYT: HSP65-6P277- and LL-pHJ: HSP65-6P277-treated groups than that in the PBS- and pCYT: Nuc-treated groups (*P* = 0.0005 for the LL-pCYT: HSP65-6P277 group vs. the PBS-treated group; *P* = 0.0008 for the LL-pHJ: HSP65-6P277 group vs. the PBS-treated group). Moreover, the levels of IFN-γ production were considerably reduced in the treatment groups, compared to those in the PBS- and pCYT: Nuc-treated groups (Fig 5B, *P* = 0.0370 for the LL-pCYT: HSP65-6P277 group vs. the PBS-treated group; *P* = 0.0255 for the LL-pHJ: HSP65-6P277 group vs. the PBS-treated group). No significant statistical difference was observed regarding the levels of IL-10 and IFN-γ production between the PBS group and the pCYT: Nuc group (*P* = 0.57 for IL-10; *P* = 0.91 for IFN-γ). These data imply that the induced P277-specific tolerance by recombinant *L. lactis* was associated with activating Th2 response and suppressing Th1 response.

## Discussion

In the current study, two novel strains of recombinant *L. lactis* (LL-pCYT: HSP65-6P277 and LL-pHJ: HSP65-6P277) delivering HSP65 and tandemly repeated P277 were constructed and tested in orally immunized NOD mice. The results showed a significantly reduction the incidence of DM1 in animals treated with either recombinant *L. lactis* strain. Further study confirmed that the treatment resulted in HSP65- and P277-specific immune tolerance as indicated by the suppression of splenocytes proliferation in response to HSP65 and P277. The DM1-preventing function could be due to an enhanced Th2-like immune response initiated by the recombinant *L. Lactis* vector. Our study suggested a potential therapeutic approach of orally administered recombinant *L. lactis* delivering HSP65-6P277 in preventing DM1.

Among current treatment options for DM1, even though normoglycemia is achievable with insulin replacement, the underlying autoimmune response that impairs and eventually eradicates beta-cells is not inhibited. Insulin replacement cannot prevent the associated complications, a major source of patient morbidity and mortality. Moreover, other strategies like beta-cell replacement still face the impediment of autoimmunity in addition to allogeneic rejection [26]. Therefore, immunotherapies that directly suppress or eliminate autoimmunity is still greatly needed. In this study, we picked up the novel peptide HSP65-6P277 that has been proven to possess efficacy in preventing the onset of DM1 in NOD mice [6], [22]. Using recombinant *L. lactis* expressing HSP65-6P277 as a platform for the delivery of anti-DM1 vaccines, we avoided a problem previously faced by researchers that HSP65-6P277 does not remain stable through the process of isolation, purification and storage.

Probiotics are an attractive choice for a DM1 intervention because of their longstanding safety record and widespread public acceptance. In addition, *L. lactis* is noninvasive and thus has less potential to trigger immune system hyper-activation or side effects from prolonged use [27], [28]. Currently, *L. lactis*-based vaccines have not been evaluated under clinical trials, however, successful vaccinations have been reported using *L. lactis*-based genetically modified micro-organisms (GMO) [29] bacterial vaccines against several diseases in mice model [14], [30], [31]. In the present study, we found that *L. lactis* vaccines via oral administration were well accepted by NOD mice. No physiological changes, like food/water intake, body weight changes were observed in comparison to the PBS control group. More importantly, recombinant *L. lactis* successfully induced the expected immune effects. Our study supports the safety and efficacy of *L. lactis*-based vaccination.

In this work, two different expression models were constructed to deliver HSP65-6P277 peptide from *L. lactis*: an inducible system that produces protein intracellularly, or a constitutive system that produces protein extracellularly. Both recombinant *L. lactis* strains delivering HSP65-6P277 greatly reduced the incidence of DM1. LL-pHJ: HSP65-6P277, which expressed protein constitutively, did not express as much peptide as the nisin-inducible LL-pCYT: HSP65-6P277 under inducing conditions. However, in an environment without nisin, such as the intestines, no HSP65-6P277 protein would be generated from LL-pCYT: HSP65-6P277 beyond what was produced during the inducing conditions in culture. Therefore, the constitutive expression of secreted HSP65-P277 by LL-pHJ: HSP65-6P277 may provide for better long-term delivery in a systemic way. This could be one reason why the LL-pHJ: HSP65-6P277-treated group exhibited a better survival curve in NOD mice (Fig 2C). We have also tested the survival time and HSP65-6P277 expression level in NOD mice after gavage feeding with the recombinant *L. lactis* strains. The results showed that HSP65-6P277 was expressed in mucous membrane of small intestine. FurthermoreNevertheless, the relevance between HSP65-6P277 antigen expression level and the degree of immune tolerance is still being investigated in our following experiments.

The potential mechanism of how HSP65-P277 peptide vaccinates against DM1 may involve a switch from the pro-inflammatory Th1 to the anti-inflammatory Th2 immune response [22]. Rabbits that are nasally immunized with the fusion protein HSP65-6P277 showed repressed T-cell proliferation and increased IL-10 production, both revealed a significant suppression of the Th1 branch and indicated, probably, a switch from Th1 to Th2 [32]. However, further cytokine studies and IgG isoform assessment should be done to determine whether it is such a switch. In our study, we determined increased IL-10 levels and decreased IFN-γ levels released by splenocytes isolated from recombinant *L. lactis*-treated groups (Fig 5). Our results are in line with previous reports that delivering a pDNA encoding IL-10 to NOD mice early in the diabetogenic response leads to a prevention of overt diabetes [33]. Another study also showed that vaccination with pDNA expressing a soluble IFN-γ receptor prevented islet infiltration and autoimmune diabetes induced by streptozontocin (STZ) in NOD mice [34]. In addition, ferment products by orally ingested live probiotic vaccines in the large intestine deliver an anti-inflammatory signal within the gut-associated lymphoid tissue. Meanwhile, probiotic cells will bind to other immune receptors on intestinal macrophages, inducing them to adopt an anti-inflammatory phenotype. When these macrophages migrate to the pancreatic lymph nodes, they induce the formation of regulatory T cells, which in turn inhibits autoreactive T cells that are responsible for β-cell destruction [2].

Although our current study proved a potential pharmaceutical application of recombinant *L. lactis* in preventing onset of DM1, further research is needed before it can be used clinically. First, the levels of secreted peptide produced by LL-pHJ: HSP65-6P277 was not very high in *L. lactis*. Considerable amounts of protein remained in the cytoplasm or membrane associated fraction in the bacteria cells. This defect may be due to limiting sortase in the cell [35], or the nature of the protein HSP65-6P277. Further study may focus on improving the efficiency of protein production and secretion. In addition, replacing resistance genes with other screening markers for the target *L. lactis* strains must be explored in future research.
